# The B1 Protein Guides the Biosynthesis of a Lasso Peptide

**DOI:** 10.1038/srep35604

**Published:** 2016-10-18

**Authors:** Shaozhou Zhu, Christopher D. Fage, Julian D. Hegemann, Andreas Mielcarek, Dushan Yan, Uwe Linne, Mohamed A. Marahiel

**Affiliations:** 1 Department of Chemistry/Biochemistry, LOEWE Center for Synthetic Microbiology, Philipps-Universität Marburg, Hans-Meerwein-Strasse 4, 35032 Marburg, Germany; 2 State Key Laboratory of Chemical Resources Engineering, Beijing University of Chemical Technology, Beijing, 10029, PR China

## Abstract

Lasso peptides are a class of ribosomally synthesized and post-translationally modified peptides (RiPPs) with a unique lariat knot-like fold that endows them with extraordinary stability and biologically relevant activity. However, the biosynthetic mechanism of these fascinating molecules remains largely speculative. Generally, two enzymes (B for processing and C for cyclization) are required to assemble the unusual knot-like structure. Several subsets of lasso peptide gene clusters feature a “split” B protein on separate open reading frames (B1 and B2), suggesting distinct functions for the B protein in lasso peptide biosynthesis. Herein, we provide new insights into the role of the RiPP recognition element (RRE) PadeB1, characterizing its capacity to bind the paeninodin leader peptide and deliver its peptide substrate to PadeB2 for processing.

Natural products from microorganisms represent a rich source of new chemical scaffolds. One class of natural products, referred to as ribosomally synthesized and post-translationally modified peptides (RiPPs), has grown substantially with genome sequencing efforts in the last few decades^1^,^2^. These peptides are initially translated by the ribosome as a precursor peptide (20–110 residues) consisting minimally of a leader and core peptide sequence^1^,^2^,^3^,^4^. The leader peptide is usually important for recognition by the processing enzymes, while the core peptide itself is transformed by maturation enzymes into the final natural product^4^. By this strategy, a remarkable degree of structural diversity can be introduced on a ribosomally synthesized peptide^2^,^5^,^6^.

Lasso peptides constitute one particularly intriguing family of RiPPs whose knot-like fold confers extraordinary stability and diverse bioactivities^7^,^8^,^9^. These peptides typically consist of a 7–9-residue, N-terminal macrolactam ring through which the C-terminal tail is threaded^10^,^11^,^12^,^13^,^14^,^15^. Unthreading of this entropically demanding fold is prevented by placement of sterically hindered side chains above and below the ring (Fig. 1a)^16^,^17^,^18^,^19^,^20^,^21^.

Since their discovery 25 years ago, more than 40 different lasso peptides along with some of their gene clusters have been identified from bioactivity screens or genome mining approaches^7^,^8^,^9^,^12^,^22^,^23^. In general, the gene clusters can be assigned to five clades according to biosynthetic machinery (Fig. 1b)^24^. For example, the proteobacterial peptide microcin J25 is synthesized by a pathway involving four gene products: *mcjA* encodes the precursor peptide; *mcjB*, an ATP-dependent cysteine protease; *mcj*C, an ATP-dependent asparagine synthetase homolog; and *mcjD*, an ABC transporter^25^,^26^,^27^,^28^,^29^,^30^,^31^,^32^. Another recently identified series of proteobacterial lasso peptides, including astexin-1, sphingopyxin I, and caulosegnin I, are synthesized from gene clusters that feature a lasso peptide-specific isopeptidase instead of an ABC transporter^12^,^15^,^18^,^19^,^33^,^34^,^35^,^36^,^37^. Yet another putative, proteobacterial gene cluster features a lasso peptide-tailoring kinase (K) with A B1 B2 C K D organization^19^,^24^,^38^. Here, the “split” B1 and B2 proteins share homology with the N- and C-terminal domains of an “intact” B protein, respectively. Split B proteins were also observed in actinobacterial clusters such as those producing lariatin, sviceucin, and streptomonomicin (A C B1 B2 D organization) as well as firmicute clusters (C A K B1 B2 D organization) such as that of paeninodin^11^,^13^,^39^,^40^. Kinases from the latter were recently characterized as tailoring enzymes of lasso peptide precursors, ultimately yielding phosphorylated lasso peptides^24^,^38^. A general schematic for the proposed route of lasso peptide biosynthesis is presented in Fig. 1c.

While *in vivo* studies have been extensively conducted on lasso peptides, there are few reports of *in vitro* experiments, each focusing on microcin J25^26^,^27^,^28^,^32^,^41^. In the latter, McjB and McjC were found to catalyze formation of the lasso fold interdependently, suggesting the necessity of a protein complex^32^. Based on the unique, threaded topology of the molecule, it was proposed that the precursor peptide prefolds into a product-like conformation prior to McjC-catalyzed closure of the macrolactam ring. A recent study showed that the B1 protein StmE serves as an RRE that binds the streptomonomicin leader peptide StmA with nanomolar affinity, presumably for delivery to the maturation machinery^42^. However, further analysis of StmE was not pursued, and no B2 fragment has been characterized.

Herein, the biosynthetic machinery of paeninodin was employed to probe the role of PadeB1 in lasso peptide biosynthesis. We first demonstrate that artificial splitting of intact B proteins and fusing of split B proteins does not abolish *in vivo* lasso peptide production. Isothermal titration calorimetry (ITC) was then utilized to determine a nanomolar binding affinity between PadeB1 and the leader peptide PadeA. Furthermore, the leader peptide binding site was mapped on PadeB1 by hydrogen-deuterium exchange mass spectrometry (HDX). These results provide a structural basis for the interaction, as supported by mutational analysis of specific PadeB1 residues. Lastly, *in vitro* experiments clearly illustrate that the protease activity of PadeB2 depends on the presence of PadeB1. We propose that formation of a macromolecular complex is necessary for the proper function of these enzymes. Taken together, our findings contribute to the currently limited understanding of lasso peptide biosynthesis and set the stage for further *in vitro* studies of this peculiar class of natural products.

## Results

### Splitting and fusing of B proteins in lasso peptide biosynthesis is reversible

In many cases, the two domains of B proteins from lasso peptide biosynthetic machineries have been observed on separate open reading frames^13^,^17^,^24^,^39^,^40^,^42^. Past research showed that, in general, the B protein contains two conserved motifs: one at the N-terminus and one at the C-terminus^19^. In all proteobacterial systems characterized thus far, the B protein is intact, while in the clusters producing lariatin, lassomycin, streptomonomicin, sviceucin and paeninodin it is split^13^,^17^,^24^,^39^,^40^. Whether the intact B protein evolved from fusion of two genes or the split B protein from division of one is unknown^7^,^35^. To examine whether machineries with artificially split or fused B proteins remain active, we first fused the split B protein of the paeninodin system by site-directed, ligase-independent mutagenesis (SLIM; the kinase-encoding gene was previously deleted)^24^,^43^,^44^. To test for production of lasso peptides *in vivo*, the resulting plasmid was expressed under previously established conditions^24^. Cell pellets were extracted and analyzed via LC-FT-MS. Interestingly, fusing the B1 and B2 proteins did not affect lasso peptide yield relative to the wild type system (Fig. 2a and Supplementary Fig. S1). To test whether a B protein could function as two fragments as in the native paeninodin system, the intact B protein of the rubrivinodin system was split by introducing a stop codon, followed by a ribosomal binding site and start codon, between the domain boundaries defined by alignments with native B1 and B2 proteins^19^. The resulting system was expressed, extracted and analyzed as above^19^. As expected, the gene cluster could still produce detectable amounts of lasso peptide, although yield was significantly lower than with the wild type system (Fig. 2b and Supplementary Fig. S2). This decreased yield could be due to the limited solubility of the artificially generated B1 protein. Indeed, when the B1 fragment was heterologously expressed in *E. coli* BL21(DE3), no soluble protein was observed (Supplementary Fig. S3). These experiments show that artificially split or fused B proteins remain active, and that the process is generally reversible.

### PadeB1 binds the leader peptide with nanomolar affinity

**Figure 1:**
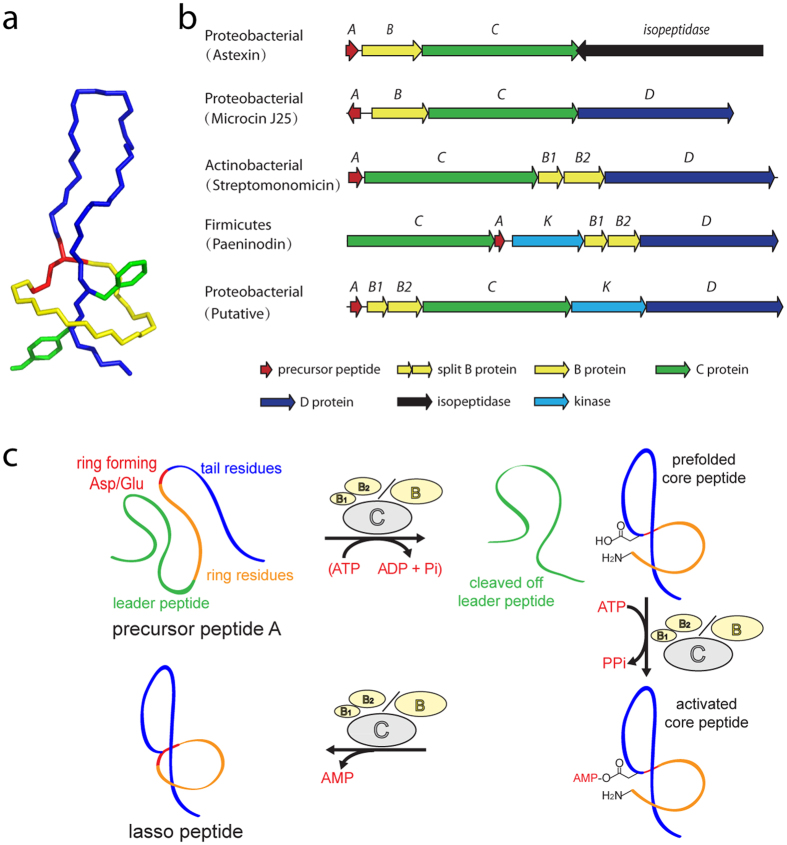
Representative structure and biosynthesis of a lasso peptide. (**a**) Peptide backbone of microcin J25 (PDB code 1Q71), colored by macrolactam ring (yellow), isopeptide bond-forming residue (red), loop and tail (blue), and plug residues (green; side chains shown). (**b**) Architecture of five different clades of lasso peptide gene clusters. (**c**) Schematic of the suggested mechanism for lasso peptide biosynthesis. ATP dependence was previously shown for McjB in the first step of microcin J25 maturation.

**Figure 2:**
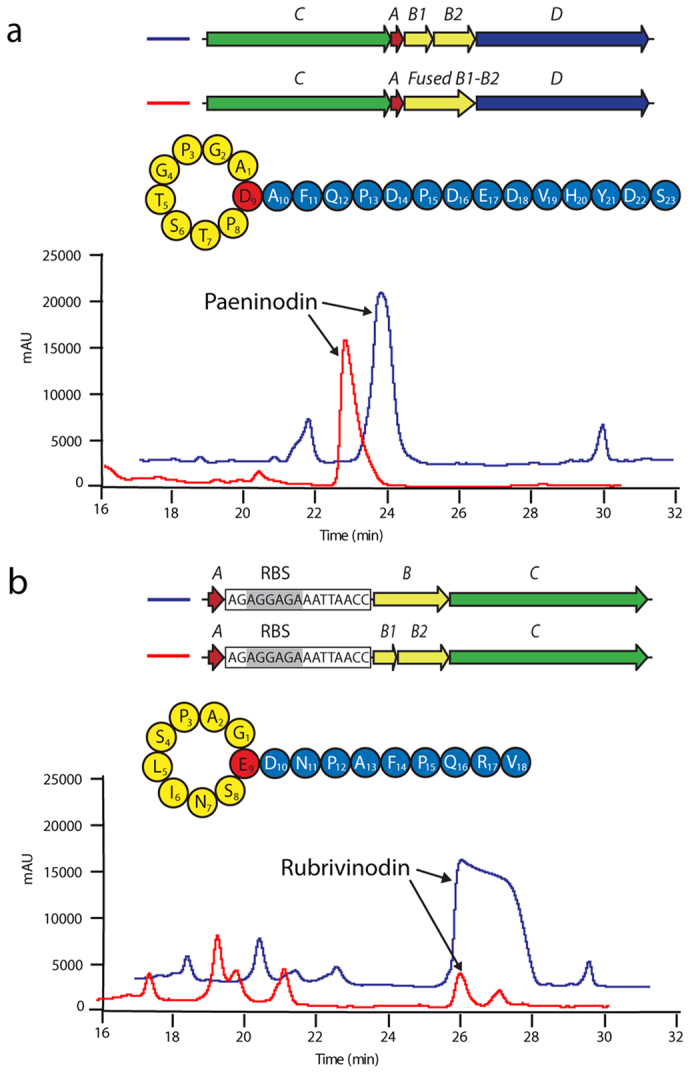
Artificially split and fused B proteins remain active. LC-MS analysis of extracts from *E. coli* cultures expressing (**a**) pET41a-*pade*CAB1B2D (control) and pET41a-*padeCA-fusedB1-B2D* (fused B) or (**b**) pET41a-*ruge*A_RBS_*BC* (control) and pET41a-*rugeA*_RBS_*B1B2C* (split B).

**Figure 3:**
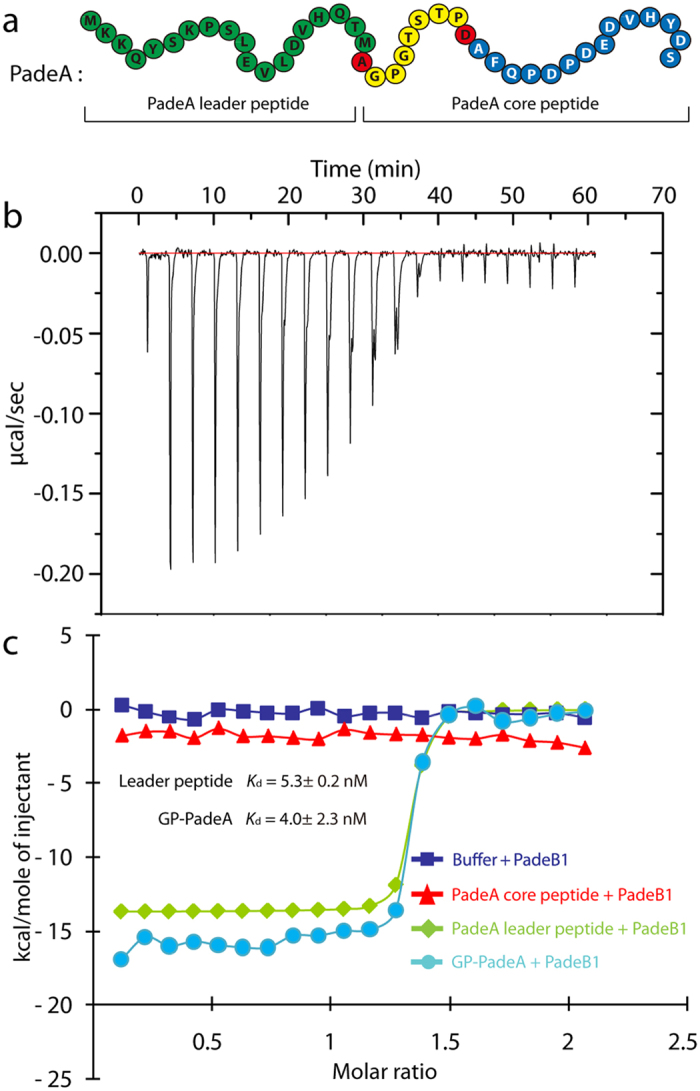
Affinity of PadeB1 for its leader peptide, as determined by ITC. (**a**) Paeninodin precursor peptide PadeA. Color code: leader peptide (green), isopeptide bond-forming residues (red), macrolactam ring (yellow), C-terminal loop and tail (blue). (**b**) Representative isothermal trace from one experiment with PadeB1 and its leader peptide. (**c**) Representative binding curve between PadeB1 and its leader peptide (green), core peptide (red), full-length precursor peptide GP-PadeA (light blue), or buffer (dark blue). The first injection for each experiment was omitted from data analysis, and the single-site model curve generated for the experimental data is shown.

B1 proteins are often annotated as homologs of the PqqD enzyme superfamily and deemed essential for lasso peptide biosynthesis^45^. A recent study revealed tight binding of RREs from several classes of RiPPs to their corresponding leader peptides, including the B1 protein StmE to its leader peptide StmA^42^. We also recently identified a new family of lasso peptide gene clusters from firmicutes that feature a B1 protein (Fig. 1b). One such protein, PadeB1 (99 residues), is involved in the biosynthesis of paeninodin^24^. To examine the B1 protein-precursor peptide interaction in this system, we cloned the PadeB1-encoding gene into a pET vector for heterologous expression and purification (Supplementary Fig. S4). The leader peptide was synthesized by Biomatik, while the core peptide and full-length precursor peptide GP-PadeA were purified as TEV protease-cleavable fusions with Trx (thioredoxin) (Fig. 3a and Supplementary Fig. S5)^46^. Each peptide was then tested for its affinity for PadeB1 by ITC (Fig. 3b,c).

In accordance with previous measurements between the B1 protein StmE and its streptomonomicin leader peptide (*K*_d_ of 35 ± 10 nM), PadeB1 was found to bind its leader peptide with a *K*_d_ of 5.3 ± 0.2 nM^42^. That only a minor difference in affinity was observed in the presence of the full-length precursor (*K*_d_ of 4.0 ± 2.3 nM) indicates that the leader region contributes most to binding PadeB1. Indeed, ITC experiments between PadeB1 and the core peptide alone failed to reveal an interaction.

### Mapping of the PadeB1-leader peptide interaction by hydrogen-deuterium exchange mass spectrometry

**Figure 4:**
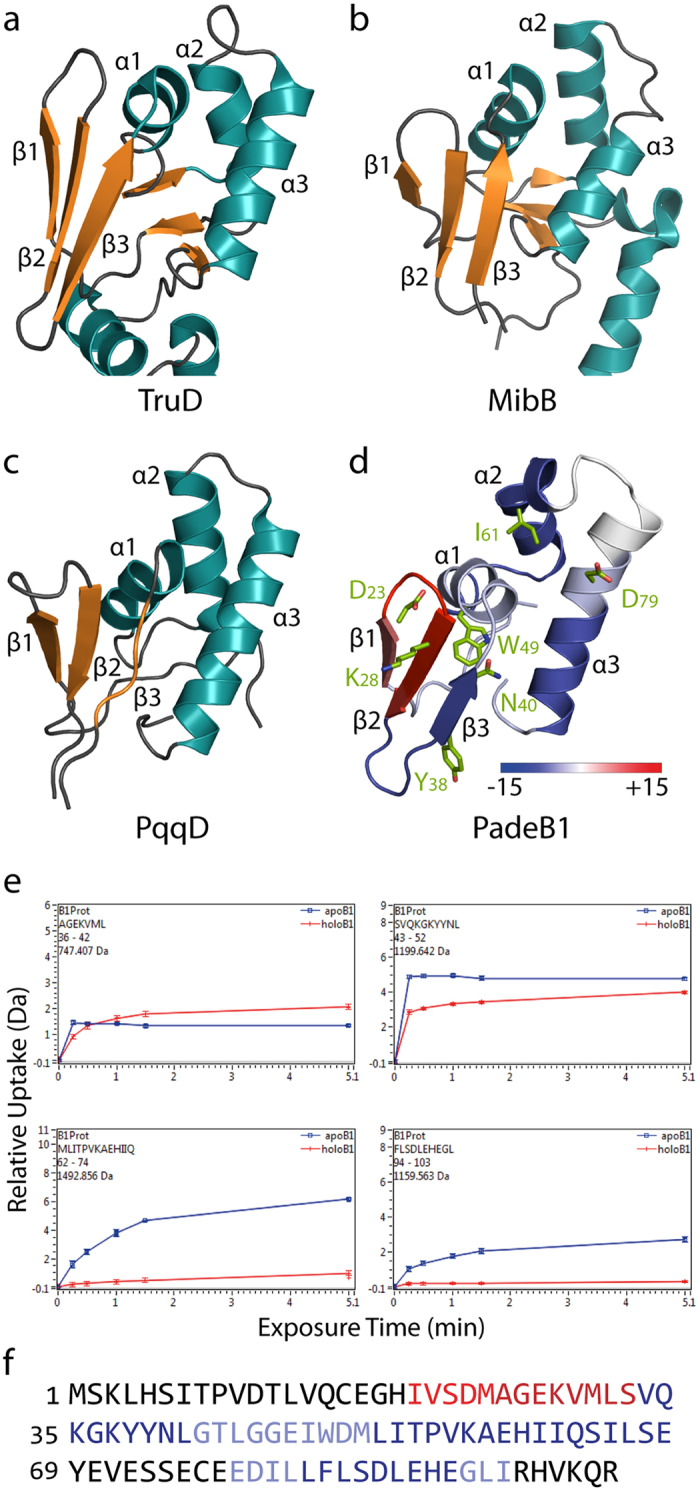
Differential HDX analysis of the PadeB1-leader peptide complex versus free PadeB1. (**a**–**c**) Structural comparison of the conserved RRE from three RiPP-modifying enzymes: (**a**) TruD, heterocyclase involved in the biosynthesis of cyanobactins (PDB code 4BS9); (**b**) MibB, dehydratase involved in the biosynthesis of the lantibiotic NAI-107 (PDB code 5EHK); and (**c**) PqqD, rSAM-associated protein involved in the biosynthesis of the bacterial dehydrogenase cofactor PQQ (PDB code 3G2B). (**d**) Mapping of regions with altered deuterium uptake on the PadeB1 homology model. A decrease in uptake (blue) signals protection (*e.g.*, a binding event), whereas an increase (red) signals exposure (*e.g.*, structural rearrangement). The colored bar indicates the percent change in hydrogen-deuterium exchange. Ala-substituted residues are shown as green sticks. (**e**) Kinetics of deuterium incorporation in four representative PadeB1 peptides, showing significant differences between leader peptide-bound PadeB1 (red) and free PadeB1 (blue). Error bars indicate mean ± s.d. of triplicate measurements. (**f**) Results from (**d**), mapped on the primary sequence of PadeB1.

To better understand how binding occurs between the B1 protein and leader peptide, we turned to hydrogen-deuterium exchange mass spectrometry (HDX). As crystals of PadeB1 with or without the leader peptide were unobtainable, a Phyre2 homology model was constructed with the corresponding domain of heterocyclase TruD (PDB code 4BS9; 23% identity), involved in biosynthesis of cyanobactins, as a template (Fig. 4a)^47^,^48^. Notably, homology to PqqD (PDB code 3G2B; 18% identity), a small protein involved in the biosynthesis of pyrroloquinoline quinone, and the C-terminal domain of MibB (PDB code 5EHK; 21% identity), a dehydratase involved in the biosynthesis of the lantibiotic NAI-107, was also predicted with high confidence (Fig. 4b–d)^49^,^50^. This was in agreement with the reported conservation of RRE domains among many different classes of RiPPs^42^,^46^.

As our ITC measurements showed no affinity between the B1 protein and the core peptide, we opted for HDX analysis of the B1 protein in the presence of the leader peptide. Generally, exposure of PadeB1 to deuterated buffer induces rapid exchange between amide hydrogens in flexible or exposed regions and deuterium in the solvent. Binding of the leader peptide, however, protects specific sites from isotopic exchange, which is readily detectable by MS. In these experiments, PadeB1 (50 μM) alone or with 100 μM leader peptide was exchanged into deuterated buffer. Exchange was halted by the addition of quenching buffer and the samples were analyzed by high-resolution MS.

Subsequent differential HDX comparing leader peptide-bound PadeB1 *versus* free PadeB1 revealed a candidate binding site for the peptide on a model (Supplementary Fig. S6). Interestingly, almost every region of PadeB1 was affected by the interaction, indicating significant conformational rearrangements. As illustrated in Fig. 4d, an increase in hydrogen-deuterium exchange (red) was observed for the N-terminal β1 and β2 strands, indicating that they became more flexible or exposed after binding. These conformational changes may be necessary for interaction with the downstream maturation machinery after precursor peptide binding. Conversely, helices α1, α2, and α3 as well as strand β3 exhibited decreased hydrogen-deuterium exchange (blue) and therefore became protected upon binding. These results support a binding model in agreement with that observed for other RREs: the leader peptide lies along α3 while interacting with β3 to complete a four-stranded β-sheet (Supplementary Fig. S7)^42^. Peptide binding evidently leads to stabilization of the protein core, as evidenced by reduced hydrogen-deuterium exchange in α1 and α2. Such rearrangement may be important for tight binding of leader peptides by RREs or for interaction with the downstream maturation machinery.

To validate our HDX-based model, we prepared the following variants and tested their effect on the PadeB1-leader peptide interaction: D23A, K28A, Y38A, N40A, W49A, I61A, and D79A (Fig. 4d). All variants were generated by SLIM, expressed and purified (Supplementary Fig. S8), and assessed for binding by ITC (Supplementary Fig. S9). Binding of the leader peptide to the D23A, K28A, Y38A variant was not perturbed; however, affinity for variants N40A, W49A, I61A, and D79A was diminished to varying degrees (Table 1). In our PadeB1 homology model, each of these residues faces “inward” toward the protein core (except for D23A and K28A) and resides on a different secondary structural element (except for Y38 and N40). Disruption of this core presumably hinders binding of the leader peptide and/or the ability of the B1 fragment to properly stabilize after binding. In summary, these experiments not only provide structural evidence for a B1 protein-leader peptide binding mode bearing semblance to that known for other RREs, but also allow insight into the conformational dynamics of a B1 protein following leader peptide binding^42^.

### The B1 fragment presents the leader peptide to B2 fragment for cleavage

**Figure 5:**
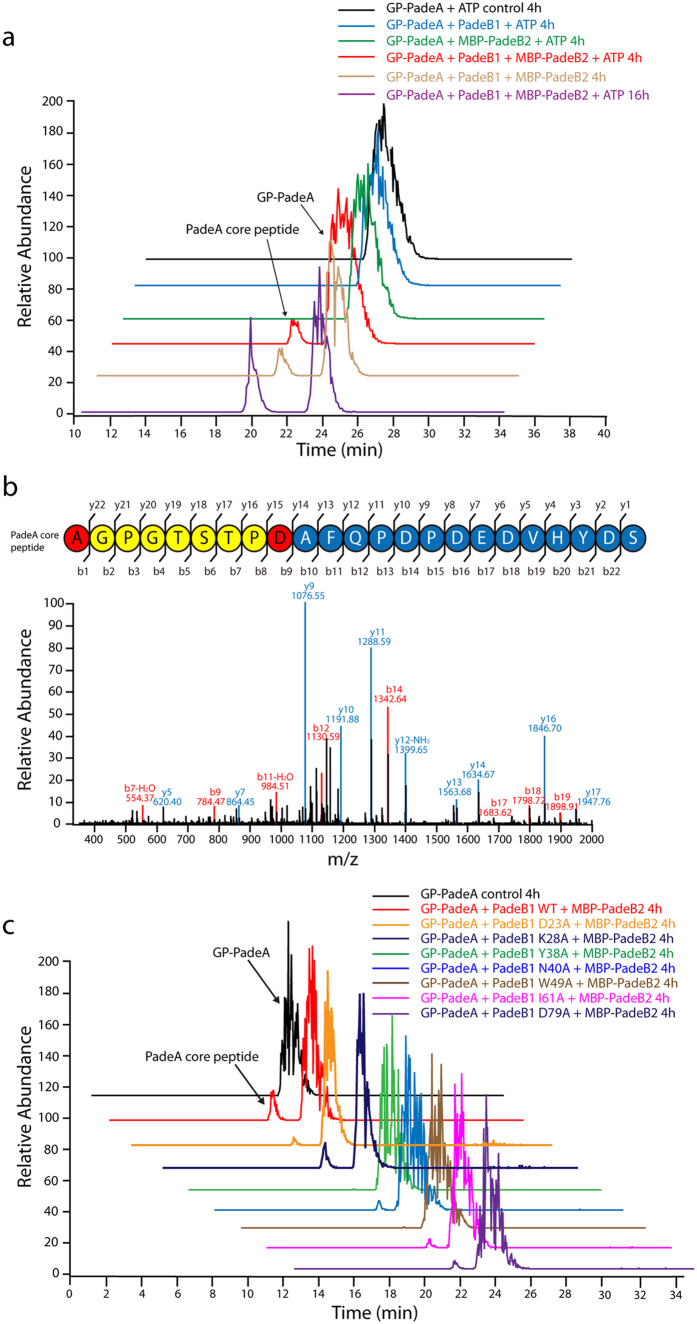
*In vitro* cleavage of the PadeB1-bound precursor peptide by PadeB2, as monitored by LC-MS. (**a**) Extracted ion chromatograms showing the [M + 5H]^5+^ ion (m/z = 963.85) of the precursor peptide and [M + 2H]^2+^ ion (m/z = 1209.50) of the core peptide. The core peptide appears only when both PadeB1 and MBP-PadeB2 are present. (**b**) MS^2^ spectrum of the core peptide obtained from assays in (**a**). The primary sequence of the core peptide is illustrated above. Color code: isopeptide bond-forming residues (red), macrolactam ring (yellow), C-terminal loop and tail (blue). (**c**) Extracted ion chromatograms from cleavage assays with PadeB1 variants, MBP-PadeB2, and GP-PadeA.

To demonstrate that PadeB1 can properly present the precursor peptide to other maturation enzymes after the binding, *in vitro* protease assays were performed with MBP-PadeB2. An MBP-PadeB2 fusion was then heterologously expressed in *E. coli* BL21(DE3) and purified to homogeneity (the protein was largely insoluble in the absence of an MBP tag) (Supplementary Fig. S10). A standard reaction (50 μL) containing Tris•HCl (50 mM, pH 8.0), MgCl_2_ (5 mM), ATP (1 mM), GP-PadeA substrate (5 μM), and PadeB1 (5 μM) and/or MBP-PadeB2 (5 μM) was performed at room temperature. Changes in mass as a result of cleavage of GP-PadeA were monitored by LC-MS. As expected, each B protein fragment failed to hydrolyze the precursor peptide in the absence of the other. However, when the precursor peptide was incubated with both PadeB1 and MBP-PadeB2, a new peak appeared (Fig. 5a). This peak contained a doubly-charged ion with m/z = 1209.5, as expected for the core peptide resulting from precursor peptide hydrolysis. Collision-induced dissociation of the ion produced a series of consecutive b- and y-fragments matching those predicted for the core peptide (Fig. 5b and Supplementary Fig. S11). The caulonodin I precursor peptide S-CnA1 was tested as an alternate substrate to investigate the specificity of the maturation machinery. As expected, the B1 and B2 fragments were unable to cleave S-CnA1, demonstrating specificity for the native precursor peptide (Supplementary Fig. S12).

Next, protease assays were carried out with the PadeB1 variants to test whether proper substrate presentation impacts processing by PadeB2 (Fig. 5c). Indeed, when most of the variants were incubated with MBP-PadeB2 and the precursor peptide, no or significantly less core peptide accumulated than with WT PadeB1. Only the K28A variant produced similar amounts of core peptide as WT PadeB1, in line with its undiminished affinity for the leader peptide. Interestingly, while the D23A and Y38A variants bound the leader peptide with near-WT affinity, very little or no conversion of PadeA was observed in their presence. Impairment of a PadeB1-PadeB2 interface by D23A and Y38A exchanges could explain the sharp decline in precursor peptide cleavage observed here. As D23, Y38, and K28 are positioned on adjacent β-strands, we hypothesize that D23 and Y38 are important in maintaining a binding surface, while K28 serves a non-crucial role. Further studies will be necessary to clarify the precise nature of a B1-B2 interaction.

These assays highlight the importance of efficient substrate recognition by PadeB1 for subsequent cleavage. Namely, all PadeB1 variants that exhibited significantly lower affinity for the leader peptide also exhibited a much lower rate of cleavage in assays with MBP-PadeB2 and PadeA. Our findings are in line with previous studies of other RiPP systems that illustrate the necessity of an RRE for precursor peptide processing^46^,^51^. Taken together, our experiments shed light on the dual functions of B1 proteins: 1) highly selective substrate recognition and 2) interaction with dedicated processing enzymes, combined with delivery of substrate to the appropriate active sites. While the precise mode of substrate transfer remains elusive, the general site of interaction may involve the C-terminal region of PadeB1.

Surprisingly, when ATP was excluded from protease assays of the B1 and B2 fragments, we continued to observe cleavage of the precursor peptide (Fig. 5a). This finding constrasts with the ATP-dependence previously observed for intact McjB^32^. Unfortunately, as PadeC was not isolatable due to insolubility, we were unable to test whether ATP is needed for proper delivery of the peptide substrate to the C protein. Nevertheless, our results clearly demonstrate that PadeB1 and PadeB2 are sufficient for precursor peptide cleavage, and that they act independently of PadeC. This also constrasts with the microcin J25 biosynthetic machinery, whose B and C proteins were suggested to function interdependently^32^. Such discrepancies may be explained by the evolutionary distance between the two systems; i.e., they originate from different phyla, contain distinct gene cluster arrangements, and differ in B protein composition (PadeB is split, MjcB is fused). Additionally, a recent study provided experimental evidence that the B and C proteins of the astexin-1 system may also function independently of one another^52^. Hence, our results indicate that not all lasso peptide processing enzymes are necessarily interdependent like McjB and McjC.

## Discussion

In the current study, we uncovered new insights into the role of the PqqD-like B1 protein in lasso peptide biosynthesis. We demonstrated the reversibility of B protein splitting and fusion, revealing that both routes are feasible from an evolutionary standpoint and that the B protein activities could be assigned to distinct domains. ITC experiments verified high-affinity leader peptide binding by PadeB1 (*K*_d_ ≈ 5 nM). Furthermore, HDX was applied to map the leader peptide binding site on the B1 protein and examine the conformational dynamics of the process, thus providing structural evidence for a physical interaction. Our data are consistent with a model in which the leader peptide lies along helix α3 while forming a fourth β-strand adjacent to β3, as previously observed in other RiPP systems^42^.

Past research suggested that the B protein is involved in prefolding the precursor peptide into a lasso-like conformation—a process which may require input of energy^32^. In the current study, we demonstrate that ATP is not required for interaction of the B1 and B2 proteins or protease activity. Indeed, our *in vitro* protease assays revealed that, after PadeB1 binds the leader region of the precursor peptide, the substrate is delivered to PadeB2 for processing. Interaction of the B1 protein with the precursor peptide was shown to be essential for downstream processing, as the B2 protein failed to cleave the substrate in the absence of the B1 fragment or in presence of B1 variants with low affinity for the leader peptide. Additionally, that protease activity was affected by β-sheet variants of PadeB1 with near-WT affinity for the leader peptide suggests a role for this region in the PadeB1-PadeB2 interface. This is in agreement with our HDX results, which revealed increased flexibility in the β1 and β2 strands upon leader peptide binding—such rearrangements may be necessary for the interaction of PadeB1 (or other RREs, in general) with downstream maturation enzymes. After processing by the B proteins, the substrate must be transferred to PadeC for macrolactam ring formation, the final step of lasso peptide biosynthesis. Unfortunately, PadeC could not be investigated due to technical reasons. However, our findings differ from those of an *in vitro* study of the microcin J25 biosynthetic machinery in which McjB-catalyzed precursor peptide cleavage was shown to be dependent on ATP and McjC^32^. The latter system may behave differently as it arises from a clade encoding an intact B protein and originates from another bacterial phylum. Further studies must address whether other clades rely on alternative strategies for lasso peptide maturation.

A number of key questions remain to be answered regarding the biosynthesis of lasso peptides. For example, the modes of interaction between the precursor peptide-bound B1 fragment and the B2 fragment or that between the B and C proteins are unknown. Structural details are also lacking for each component of the maturation machinery. Nevertheless, the current study pushes our understanding forward by demonstrating two essential features for PadeB1: leader peptide binding and substrate delivery to PadeB2. The first cleavage step performed by B1 and B2 is important in understanding the precise mechanism of lasso peptide maturation. Thus, our findings lay a foundation for future *in vitro* investigations of this unique class of natural products.

## Methods

### Strains and Materials

*Paenibacillus dendritiformis* C454, generously provided by Eshel Ben-Jacob and Alin Finkelshtein (Tel Aviv University, Israel), was used to prepare genomic DNA. Plasmid sequencing was performed by GATC Biotech. Oligonucleotides of HPLC purity were purchased from Sigma Aldrich. Isopropyl-β-d-thiogalactoside (IPTG) and kanamycin were purchased from Panreac AppliChem. Phusion High-Fidelity DNA Polymerase and Gibson Assembly Master Mix were purchased from New England Biolabs. The leader peptide of PadeA was synthesized by Biomatik (>95% purity). All other reagents were purchased from Sigma Aldrich unless otherwise specified. M9 minimal medium contained Na_2_HPO_4_·12 H_2_O (17.1 g/L), KH_2_PO_4_ (3 g/L), NaCl (0.5 g/L), NH_4_Cl (1 g/L), 2 M MgSO_4_ solution (1 mL/L), 0.5 M CaCl_2_ solution (0.2 mL/L), 40% w/v glucose solution (10 mL/L), and vitamin mix (2 mL/L added after autoclaving; see Supplementary Table S1). Kanamycin was used at a concentration of 50 μg/mL.

### Splitting and Fusing of B Proteins in the Paeninodin and Rubrivinodin Biosynthetic Gene Clusters

Mutagenesis was achieved by site-directed ligation-independent mutagenesis (SLIM) with pET41a-*padeCAB1B2D* and pET41a-*rugeA*_RBS_*BC* (prepared previously) as PCR templates, respectively^19^,^24^,^43^,^44^. Generally, the resulting PCR products (one generated with primers P1 and P4 and one with P2 and P3; Supplementary Table S2) were digested with *Dpn*I for 2 h at 37 °C, inactivated for 20 min at 80 °C, and combined for hybridization according to the SLIM protocol. Product plasmids, isolated from individual *E. coli* TOP10 transformants, were then retransformed into *E. coli* BL21(DE3) cells. An overnight culture in LB medium was used to inoculate 10 × 500 mL M9 minimal medium with kanamycin in 2-L flasks and grown at 37 °C with shaking. At OD_600_ ≈ 0.6–0.8, IPTG (0.05 mM) was added to induce expression. Cells were cultivated at 37 °C for 1 day (rubrivinodin) or 3 days (paeninodin), in accordance with previously established conditions^19^,^24^. After centrifugation (8000 rpm, 20 min, 4 °C), pellets were vortexed and mixed with 60 mL MeOH for overnight extraction at 4 °C with shaking. Following centrifugation (6000 rpm, 20 min, 4 °C), the pellet extract was filtered and solvent was evaporated at 40 °C and reduced pressure. Dried extracts were resuspended in 900 μL 50% MeOH, clarified by centrifugation (12000 rpm, 60 min), and analyzed via LC-FTMS (see below).

### Mass Spectrometric Analysis of Culture Extracts

LC-FTMS of culture extracts (100 μL per injection) was performed on a high-resolution LTQ-FT Ultra mass spectrometer (Thermo Fisher Scientific) connected to an Agilent 1100 HPLC system with a NUCLEODUR 100-3 C_18_ ec column (125 mm × 2 mm; Macherey-Nagel). The following gradient of water/0.1% TFA (solvent A) and MeCN/0.1% TFA (solvent B) was applied at a flow rate of 0.2 mL/min: isocratic 2% B for 2 min, linear increase from 2–30% B for 18 min, linear increase from 30–95% B for 15 min, and isocratic 95% B for 2 min. To fragment the selected masses, collision-induced dissociation fragmentation was performed within the linear ion trap. Different charged ions were selected for fragmentation based on their predominance. The energy of fragmentation was set to 35% for each measurement.

### Cloning, Expression, and Purification of PadeB1 and MBP-PadeB2

Whole genomic DNA of *P. dendritiformis* C454 was prepared using a standard protocol (FastDNA SPIN Kit, MP Biomedicals). The genes *padeB1* (GenBank accession: WP_006678398) and *padeB2* (GenBank accession: EHQ60563.1) were amplified from this template (see Supplementary Table S3 for primers). The backbone of a pET MBP-1a vector in which the TEV protease site was previously exchanged with an HRV 3C site was PCR-amplified for cloning by Gibson assembly (see Supplementary Table S3 for primers)^24^. After digesting with *Dpn*I, the solution was combined with PCR-amplified gene fragments and Gibson Assembly Master Mix according to the manufacturer’s protocol. Ligated plasmids were transformed into *E. coli* TOP10 cells and reisolated from individual transformants.

PadeB1 and MBP-PadeB2 were purified according to the same protocol. Generally, *E. coli* BL21(DE3) cells were transformed with the appropriate plasmids and grown overnight in LB medium with kanamycin at 37 °C with shaking. This culture was used to inoculate 10 × 500 mL LB medium with kanamycin in 2-L flasks and grown at 37 °C with shaking. At OD_600_ ≈ 0.6–0.8, the temperature was reduced to 18 °C, and then expression was induced by addition of IPTG (0.05 mM). After 16 h, cells were harvested by centrifugation (8000 rpm, 20 min, 4 °C), resuspended in HEPES buffer A (50 mM HEPES, 300 mM NaCl, 30 mM imidazole, 5% glycerol; pH 8.0) supplemented with lysozyme and DNase, and lysed with a French Press (SLM Aminco). Insoluble cellular debris were then removed by centrifugation (17000 rpm, 45 min, 4 °C), and the supernatant was filtered with a Filtropur S filter (0.2 μm; Sarstedt) before loading onto a 1-mL Ni-NTA column equilibrated with HEPES buffer A. After washing the column with HEPES buffer A, protein was eluted with increasing concentrations of imidazole in HEPES buffer A. Fractions containing the target protein, as identified by SDS-PAGE, were pooled and concentrated in an Amicon Ultra-15 Centrifugal Filter Unit. Another round of purification by size-exclusion chromatography was performed on a HiLoad16/60 Superdex 200 pg column (GE Healthcare Life Sciences) equilibrated with HEPES buffer B (10 mM HEPES, 150 mM NaCl, 5% glycerol; pH 7.5). Pure protein fractions were identified by SDS-PAGE, concentrated, and flash-frozen before storing at −80 °C.

### Generation of PadeB1 Variants

Alanine exchanges of PadeB1 were generated by SLIM with the pET-*padeB1* plasmid as template (see Supplementary Table S4 for primers). PCR products (one generated with primers P1 and P4, the other with P2 and P3) were digested with *Dpn*I for 2 h at 37 °C, inactivated for 20 min at 80 °C, and mixed for further treatment according to the SLIM protocol^43^,^44^. Mutant plasmids, isolated from individual *E. coli* TOP10 transformants, were then retransformed into *E. coli* BL21(DE3) cells for subsequent expression. Expression and purification were carried out with the same protocol as for pET-*padeB1*.

### Cloning, Expression, and Purification of Precursor Peptides GP-PadeA and S-CnA1 and the Core Peptide of PadeA

GP-PadeA and the core peptide of PadeA were generated as described previously^24^. Purification of the caulonodin precursor peptide S-CnA1 was facilitated by fusing it to a thioredoxin (Trx) tag. The gene was PCR-amplified from *Caulobacter sp*. K31 genomic DNA using primers CnA1-FP and CnA1-RP (Supplementary Table S5)^19^. The backbone of a pET-48b(+) vector in which the HRV 3C protease site was previously exchanged with a TEV site was PCR-amplified using pET48b-FP and pET48b-RP as primers (Supplementary Table S4)^24^. The resulting amplicon was treated with *Dpn*I and ligated with the insert by Gibson assembly. Trx-CnA1 was heterologously expressed using the same method as for PadeB1. The Trx tag was cleaved by incubating the sample with TEV protease (0.1 mg/mL) for 16 h at 4 °C. After cleavage, a Ser remained on the N-terminus of CnA1, resulting in an S-CnA1 peptide. Final purification of S-CnA1 was achieved using the same procedure as for GP-PadeA^24^. Pure peptides were then subjected to LC-MS analysis as described above.

### Isothermal Titration Calorimetry Analysis

A 100 μM stock solution of each peptide was prepared in ddH_2_O, and pure PadeB1 and variants thereof were diluted to 5–10 μM in assay buffer (50 mM Tris, 5 mM MgCl_2_; pH 8.0). Isothermal titration calorimetry analysis was then performed on a MicroCal iTC200 (Malvern) at 25 °C, 600 rpm. Enzyme solutions were loaded into the sample cell and titrated with an initial volume of 1.2 μL peptide solution, followed by 19 × 2 μL peptide solution. Each experiment was performed in triplicate. Isotherms were integrated with the Origin software package (OriginLab) and the resulting integrals were fit to the “One Set of Sites” model for non-linear least squares regression, preprogrammed into Origin (see Equation 1, where *n* = number of binding sites, *M*_*t*_ = total protein concentration, *X*_*t*_ = total ligand concentration, *V*_*o*_ = cell volume, *K* = binding constant, and *ΔH* = binding enthalpy).


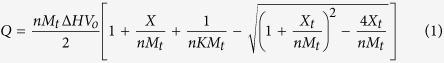


### Hydrogen-Deuterium Exchange Mass Spectrometry (HDX)

HDX experiments were conducted on a fully automated HDX PAL robot coupled to a custom-built LC-MS equipped with a pepsin column, as described previously^53^,^54^,^55^. PadeB1 (50 μM) alone or with 100 μM leader peptide was prepared in Tris buffer W (50 mM Tris, 5 mM MgCl_2_; pH 8.0). Samples were diluted ~1:50 in D_2_O exchange buffer (20 mM HEPES, 200 mM NaCl, 20 mM KCl, 20 mM MgCl_2_; pH 7.5; prepared in pure D_2_O) to a final volume of 50 μL and incubated for 15, 30, 60, and 300 s at 37 °C. PadeB1 samples in water-based buffer served as 0 s controls. Exchange reactions were stopped by addition of 50 μL quenching buffer (400 mM KH_2_PO_4_/H_3_PO_4_; pH 2.2) and injected directly into the LC-MS unit. Experiments were carried out in duplicate. After measurements, full-scan LC-MS data were acquired in triplicate. Peptides with suitable signal intensity were then identified, extracted from all HDX LC-MS data files, and saved as the peptide set for analysis. The centroid mass of each isotopic envelope (*m*) and the percent of deuterium (*D%*) were calculated by HDX WorkBench using Eq. 2. Differential HDX analysis was then performed by HDX WorkBench^56^. Differential *D%* values (*ΔD%*) were calculated for each peptide pair. Differential HDX maps were then generated by consolidating all peptide-level information and mapped onto the homology model of PadeB1.


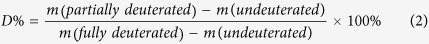


### *In vitro* Protease Assays

In a typical protease assay, PadeB1 or MBP-PadeB2 (~5 μM) was incubated with 50 μM peptide in 50 μL assay buffer (50 mM Tris, 5 mM MgCl_2_, 1 mM ATP; pH 8.0) at 37 °C for 24 h. Reactions were quenched by addition of 5% (v/v) TFA and subjected to LC-FTMS analysis as described above.

## Additional Information

**How to cite this article**: Zhu, S. *et al*. The B1 Protein Guides the Biosynthesis of a Lasso Peptide. *Sci. Rep.* **6**, 35604; doi: 10.1038/srep35604 (2016).

## Supplementary Material

Supplementary Information

## Figures and Tables

**Table 1:** Affinity of PadeB1 variants for the leader peptide.

PadeB1 variant	*K*_d_ (nM)	Site of mutation
WT	5.3 ± 0.2	—
D23A	2.4 ± 1.7	β1
K28A	2.7 ± 1.4	β2
Y38A	6.2 ± 3.3	β3
N40A	16 ± 7.9	β3
W49A	140 ± 49	α1
I61A	73 ± 33	α2
D79A	150 ± 18	α3

Errors represent mean ± s.d. of triplicate measurements.
