# Supplementary material for: The B1 Protein Guides the Biosynthesis of a Lasso Peptide
Source: Sci Rep. 2016 Oct 18;6:35604. doi: 10.1038/srep35604 (PMC5067487; doi:10.1038/srep35604)
Supplement: Supplementary Information [file srep35604-s1.pdf]

The B1 Protein Guides the Biosynthesis of a Lasso Peptide

Shaozhou Zhu[1,2], Christopher D. Fage[1], Julian D. Hegemann[1], Andreas Mielcarek[1], Dushan Yan[1], Uwe Linne[1] & Mohamed A. Marahiel*[,1]

[1]Department of Chemistry/Biochemistry, LOEWE Center for Synthetic Microbiology, Philipps-Universität Marburg, Hans-Meerwein-Strasse 4, 35032 Marburg, Germany

[2]State Key Laboratory of Chemical Resources Engineering, Beijing University of Chemical Technology, Beijing, 10029, PR China

*corresponding author: marahiel@staff.uni-marburg.de

Supplementary Table S1. M9 vitamin mix.

component	amount
choline chloride	1.0 g
folic acid	1.0 g
pantothenic acid	1.0 g
nicotinamide	1.0 g
myo-inositol	2.0 g
pyridoxal hydrochloride	1.0 g
thiamine	1.0 g
riboflavin	0.1 g
disodium adenosine 5'-triphosphate	0.3 g
biotin	0.2 g
	add 300 mL ddH_2O[a]

[a]Prior to bringing the solution to volume, 10 M NaOH was slowly added until all components were dissolved at a pH of ~12. Afterwards, the clear, orange solution was sterile-filtered and stored at 4 °C (short-term) or -20 °C (long-term).

Supplementary Table S2. SLIM primers for splitting and fusing of B proteins in the *padeCAB1B2D* and *rugeA_RBS_BC* production constructs. Plasmids pET41a-*padeCAKB1B2D* and pET41a-*rugeA_RBS_BC* were used as PCR templates, respectively. Overhang regions are underlined.

construct	name	sequence
pET41a-*padeCA-FusedB1-B2D*	PadeFusedB1B2_P1	ATG CCG TAT CAA ACC CTC ATG TTC CAA ATC CGA GAG GAA C
	PadeFusedB1B2_P2	AGA TGG ATA TGG TTA TGC TTG TTT GAC CGG AAG ACA TTC CTT TTA TTT G
	PadeFusedB1B2_P3	TCTCAACCGGTTTCG TTG CTT CAC ATG CCG TAT CAA ACC CTC ATG TTC C
	PadeFusedB1B2_P4	GTGAAGCAACGAAAC CGG TTG AGA AGA TGG ATA TGG TTA TGC TTG TTT GAC CGG
pET41a-*rugeA_RBS_B1B2C*	RugeSplitB_P1	GCT GAC GAT CGC GTC GTC CGT GTC GAG CG
	RugeSplitB_P2	TGC GCA CCA AGG ACG TTG GCC GCT TCA TCC AC
	RugeSplitB_P3	TGGTTAATTTCTCCTCTTCAGCT GAC GAT CGC GTC GTC CGT GTC GAG CG
	RugeSplitB_P4	TGAAGAGGAGAAATTAACCATGC GCA CCA AGG ACG TTG GCC GCT TCA TCC AC

Supplementary Table S3. Primers for Gibson assembly of the *padeB1 and padeB2* genes. Overhang regions are underlined.

construct	name	sequence
pET-*padeB1*	PadeB1-FP	ATCACCATCACGGCGCCCATATG AGC AAA CTT CAT TCG ATC ACC CCT GTC GAT ACG
	PadeB2-RP	TGGTGGTGGTGGTGCTCGAGTCA TCG TTG CTT CAC ATG CCG TAT CAA ACC CTC ATG
	pETMBPB1-FP	CTC GAG CAC CACCACCACCACCAC TGA GAT CCG GC
	pETMBPB1-RP	ATG GGC GCC GTG ATG GTG ATG GTG ATG TTT CAT GGT ATA TCT C
pETMBP-*padeB2*	PadeB2-FP	TTCAGGGACCCGGCGCCCAT ATGT TTG ACC GGA AGA CAT TCC TTT TAT TTG CGG AAG CTT TTC
	PadeB2-RP	TGGTGGTGGTGGTGCTCGAG TCA TGA GTC TGT CCC TGC GCT CTT CGC GAA TTT C
	pETMBPB2-FP	CTC GAG CAC CACCACCACCACCAC TGA GAT CCG GC
	pETMBPB2-FP	ATG GGC GCC GGG TCC CTG AAA GAG GAC TTC AAG AG

Supplementary Table S4. Primers for mutagenesis of *padeB1*. All mutations were introduced using SLIM. pET-*padeB1* was used as a template for all PCR reactions. Overhang regions are underlined.

construct	name	sequence
pET-*padeB1-D23A*	adeB1-D23A_P1	GCT AAC GAT ATG GCC CTC GCA TTG AAC AAG CGT ATC
	PadeB1-D23A_P2	ATG TTA AGC GTC CAG AAG GGA AAA TAC TAT AAT CTC GGT ACG C
	PadeB1-D23A_P3	CACTTTTTCGCCGGCCATCGCGCT AAC GAT ATG GCC CTC GCA TTG AAC AAG CGT ATC
	PadeB1-D23A_P4	GCGATGGCCGGCGAAAAAGTGATG TTA AGC GTC CAG AAG
pET-*padeB1-K28A*	PadeB1-K28A_P1	GCT AAC GAT ATG GCC CTC GCA TTG AAC AAG CGT ATC
	PadeB1-K28A_P2	ATG TTA AGC GTC CAG AAG GGA AAA TAC TAT AAT CTC GGT ACG C
	PadeB1-K28A_P3	CACCGCTTC GCC GGC CAT ATC GCT AAC GAT ATG GCC CTC
	PadeB1-K28A_P4	GATATGGCCGGCGAAGCGGTGATG TTA AGC GTC CAG AAG GGA AAA TAC TAT AAT CTC GGT AC
pET-*padeB1-Y38A*	PadeB1-Y38A_P1	CTT CTG GAC GCT TAA CAT CAC TTT TTC GCC G
	PadeB1-Y38A_P2	ACG CTT GGC GGC GAG ATC TGG GAC
	PadeB1-Y38A_P3	ACCGAGATTATACGC TTT TCC CTT CTG GAC GCT TAA CAT CAC TTT TTC GC
	PadeB1-Y38A_P4	GGAAAAGCGTAT AAT CTC GGT ACG CTT GGC GGC GAG ATC
pET-*padeB1-N40A*	PadeB1-N40A_P1	CTT CTG GAC GCT TAA CAT CAC TTT TTC GCC G
	PadeB1-N40A_P2	ACG CTT GGC GGC GAG ATC TGG GAC
	PadeB1-N40A_P3	ACCGAGCGCATA GTA TTT TCC CTT CTG GAC GCT TAA CAT CAC TTT TTC G
	PadeB1-N40A_P4	GGAAAATACTATGCG CTC GGT ACG CTT GGC GGC GAG ATC TG
pET-*padeB1-W49A*	PadeB1-W49A_P1	GCC AAG CGT ACC GAG ATT ATA GTA TTT TCC CTT CTG GAC G
	PadeB1-W49A_P2	ATC ACG CCC GTG AAG GCG GAA CAC ATT ATT CAA TCC ATT TTA TC
	PadeB1-W49A_P3	AAGCATGTCCGCGATCTCGCCGCC AAG CGT ACC GAG ATT ATA GTA TTT TCC CTT C
	PadeB1-W49A_ P4	GGCGAGATCGCGGACATGCTTATC ACG CCC GTG AAG GCG GAA CAC ATT ATT CAA TCC
pET-*padeB1-I61A*	PadeB1-I61A_P1	CCT TCA CGG GCG TGA TAA GCA TGT CCC AGA TCT CGC
	PadeB1-I61A_P2	TTT ATC CGA ATA TGA GGT GGA GTC GTC GGA ATG CGA GGA AGA C
	PadeB1-I61A_P3	ATGGATTGAATCGCGTGTTCCGCCT TCA CGG GCG TGA TAA GCA TGT CCC AGA TC
	PadeB1-I61A_P4	CGGAACACGCGATTCAATCCATTTT ATC CGA ATA TGA GGT GGA GTC GTC GGA ATG CGA G
pET-*padeB1-D79A*	PadeB1-D79A_P1	CTC GCA TTC CGA CGA CTC CAC CTC ATA TTC GGA TAA AAT GG
	PadeB1-D79A_P2	GAT TTG GAA CAT GAG GGT TTG ATA CGG CAT GTG AAG CAA CGA TGA
	PadeB1-D79A_P3	CGAGAGGAACAAGAGAATCGCTTCCTC GCA TTC CGA CGA CTC CAC CTC ATA TTC GGA TAA AAT GG
	PadeB1-D79A_P4	GAAGCGATTCTCTTGTTCCTCTCGGAT TTG GAA CAT GAG GGT TTG ATA CGG C

Supplementary Table S5. Primers for Gibson assembly of the *cnA1* gene into pET-48b(+). Overhang regions are underlined.

construct	name	sequence
pET48b-*cnA1*	CnA1-FP	<u>AGAATCTTTATTTTCAGTCT</u>ATG GAA CGG ATC GAA GAC CAC ATC GAC GAC GAA CTG
	CnA1-RP	<u>TAGGTTAATTAAGCCTCGAG</u>TTA GTC CCG GGA CAG GCC CGT GGG CTC CC
	pET48b-FP	CTC GAG GCT TAA TTA ACC TAG GCT GCT AAA CAA AGC C
	pET48b-RP	AGA CTG AAA ATA AAG ATT CTC AGC CGC GGA GTG ATG GTG

a

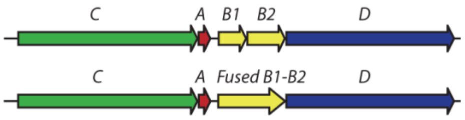

b

PadeB1:
MSKLHSITPVDTLVQCEGHIVSDMAGEKVMLSVQKGKYYNLGTLGGE
IWDMLITPVKAEHIIQSILSEYEVESSECEEDILLFLSDLEHEGLIRHVKQR

PadeB2:
LFDRKTFLLFAEAFLYLGWARILLYFPFAKISPLLGRRSMETPEVHDPSDTKT
MKHIRSAIHIASRHTFWESKCLVRAIAGMKMLEKRNLHSTLYLGTGKNKNGEL
IAHAWLRSGSIYITGAEMMKQFVVVEKFAKSAGTDS

Pade Fused B1-B2:
MSKLHSITPVDTLVQCEGHIVSDMAGEKVMLSVQKGKYYNLGTLGGE
IWDMLITPVKAEHIIQSILSEYEVESSECEEDILLFLSDLEHEGLIRHVKQR
NRLRRWIWLCLFDRKTFLLFAEAFLYLGWARILLYFPFAKISPLLGRRSM
ETPEVHDPSDTKTMKHIRSAIHIASRHTFWESKCLVRAIAGMKMLEKRN
LHSTLYLGTGKNKNGELIAHAWLRSGSIYITGAEMMKQFVVVEKFAKSA
GTDS

c

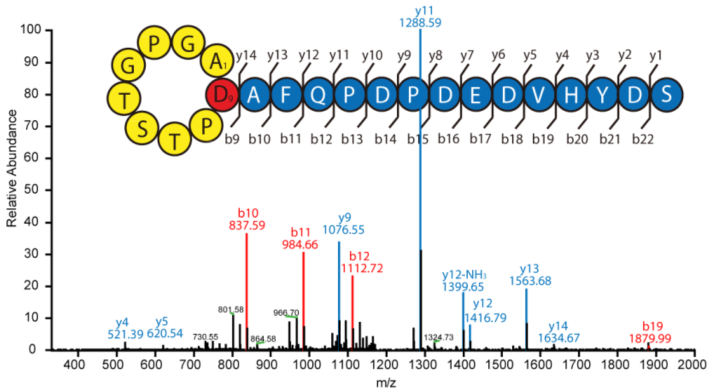

Supplementary Figure S1. (a) Schematic of gene clusters with the (top) native and (bottom) artificially fused B protein open reading frames for paeninodin biosynthesis (kinase-encoding gene deleted). (b) Sequences of native and artificially fused B proteins. The fusion was generated by a 2-bp deletion in the stop codon after the *padeB1* gene (blue), causing a frameshift that led to expression of a formerly silent, intergenic region (black) along with the *padeB2* gene (red). (c) MS^2 spectra of paeninodin from culture extracts. Color code: b-series ions (red), y-series ions (blue).

a

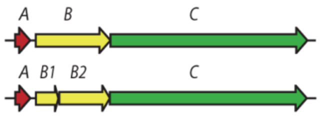

b

RugeB:
LIQPAPTSPRALSLAPHVRACATGGQVILLDLRSGTYLGLAGDRLAALAGGVAGWPNPTR
KEGETAPTSIAIYSLVRPLLARGLLVEHLVEPAPIVELEEPRSALDTDDAIVSVRTKDVG
RFIHAAGSASAWLRWHSLLWIVEAATARRGTNAKALCDFEALRHAVARYDKLRPLAFAAQ
DRCLYDSLALLLFLASEGHCPRWVVGVRTRPFAAHSWVQSGSLVLNDQAEHVRAFSPILV
V

Ruge Split B1:
MSATTRRLHPRPILIQPAPTSPRALSLAPHVRACATGGQVILLDLRSGTYLGLAGDRLAA
LAGGVAGWPNPTRKEGETAPTSIAIYSLVRPLLARGLLVEHLVEPAPIVELEEPRSALDT
DDAIVS

Ruge Split B2:
MRTKDVGRFIHAAGSASAWLRWHSLLWIVEAATARRGTNAKALCDFEALRHAVARYDKLR
PLAFAAQDRCLYDSLALLLFLASEGHCPRWVVGVRTRPFAAHSWVQSGSLVLNDQAEHVR
AFSPILVV

c

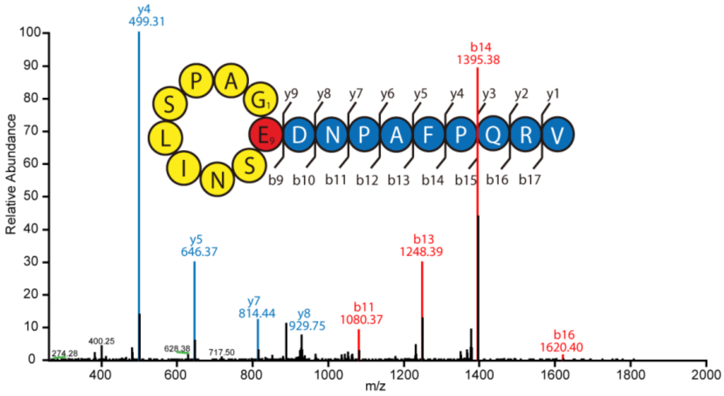

Supplementary Figure S2. (a) Schematic of gene clusters with the (top) native and (bottom) artificially split B protein open reading frames for rubrivinodin biosynthesis. (b) Sequence of original and artificially split B proteins. (c) MS^2 spectrum of rubrivinodin from culture extracts. Color code: b-series ions (red), y-series ions (blue).

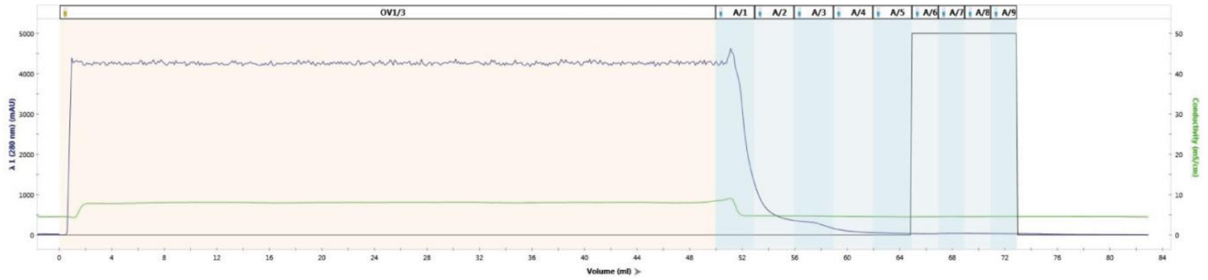

Supplementary Figure S3. UV trace (280 nm; blue) of attempted Ni-NTA purification of the artificially split B1 fragment from the rubrivinodin system. For the first ~50 mL, the lysate was applied to the column, followed by washing with HEPES buffer A (fractions A1-A5), and eluting with 200 mM imidazole in HEPES buffer A (fractions 6-9). The absence of an elution peak suggests poor solubility and/or column binding.

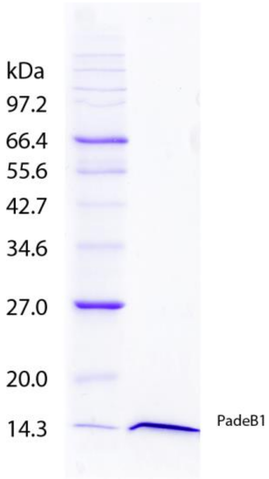

Supplementary Figure S4. SDS-PAGE gel of purified PadeB1. The protein was purified by Ni-NTA and size-exclusion chromatography.

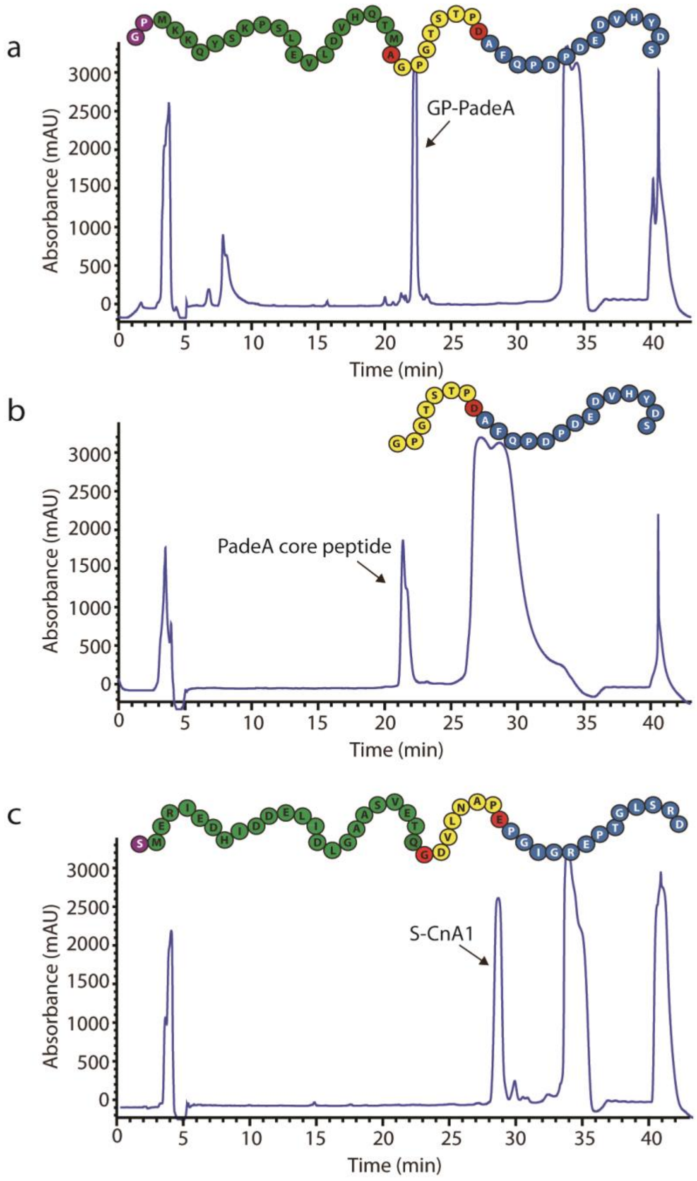

Supplementary Figure S5. Preparative HPLC chromatograms of the (a) paeninodin precursor peptide GP-PadeA, (b) core peptide of PadeA, and (c) caulonodin I precursor peptide S-CnA1.

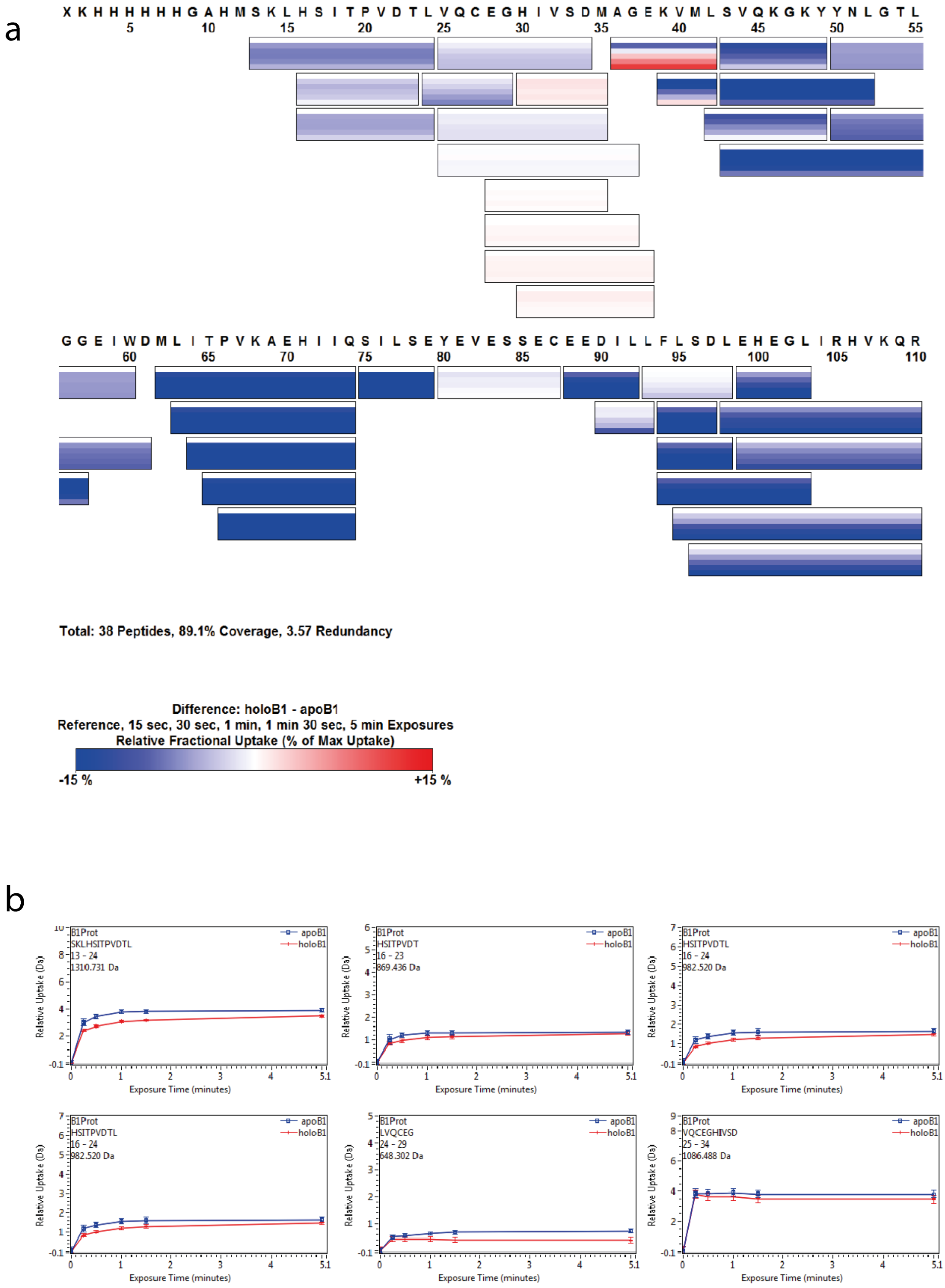

Supplementary Figure S6. (a) Differential hydrogen-deuterium exchange, mapped onto peptic peptides of PadeB1. A color guide for relative fractional uptake is shown below. (b) Kinetics of deuterium uptake for PadeB1 regions showing significant differences in leader peptidebound-PadeB1 (red) *versus* free PadeB1 (blue). Error bars represent mean ± s.d. of triplicate measurements. (Figure continues on the following page.)

b, cont.

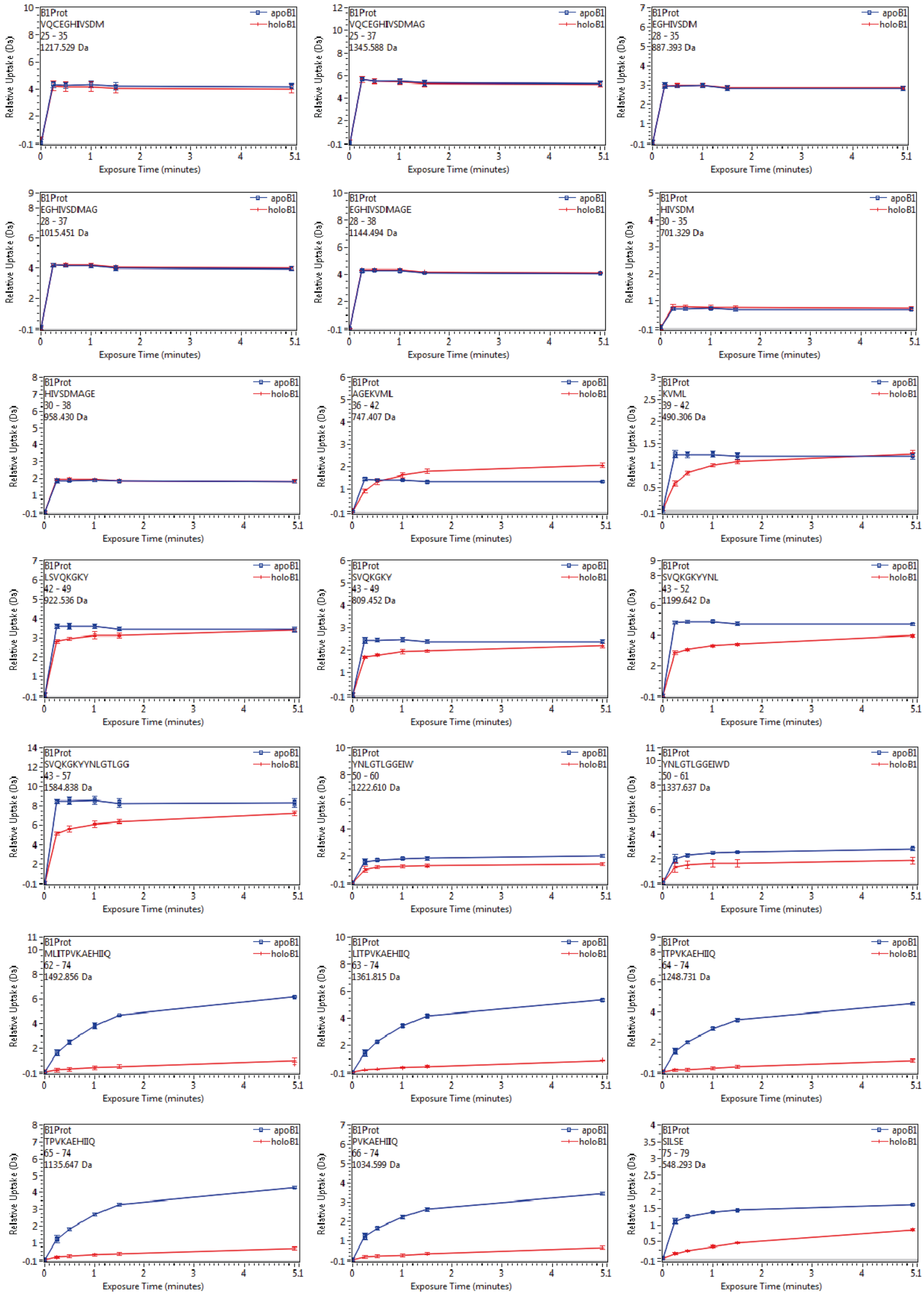

Supplementary Figure S6, cont. (a) Differential hydrogen-deuterium exchange, mapped onto peptic peptides of PadeB1. A color guide for relative fractional uptake is shown below. (b) Kinetics of deuterium uptake for PadeB1 regions showing significant differences in leader peptidebound-PadeB1 (red) *versus* free PadeB1 (blue). Error bars represent mean ± s.d. of triplicate measurements. (Figure continues on the following page.)

b, cont.

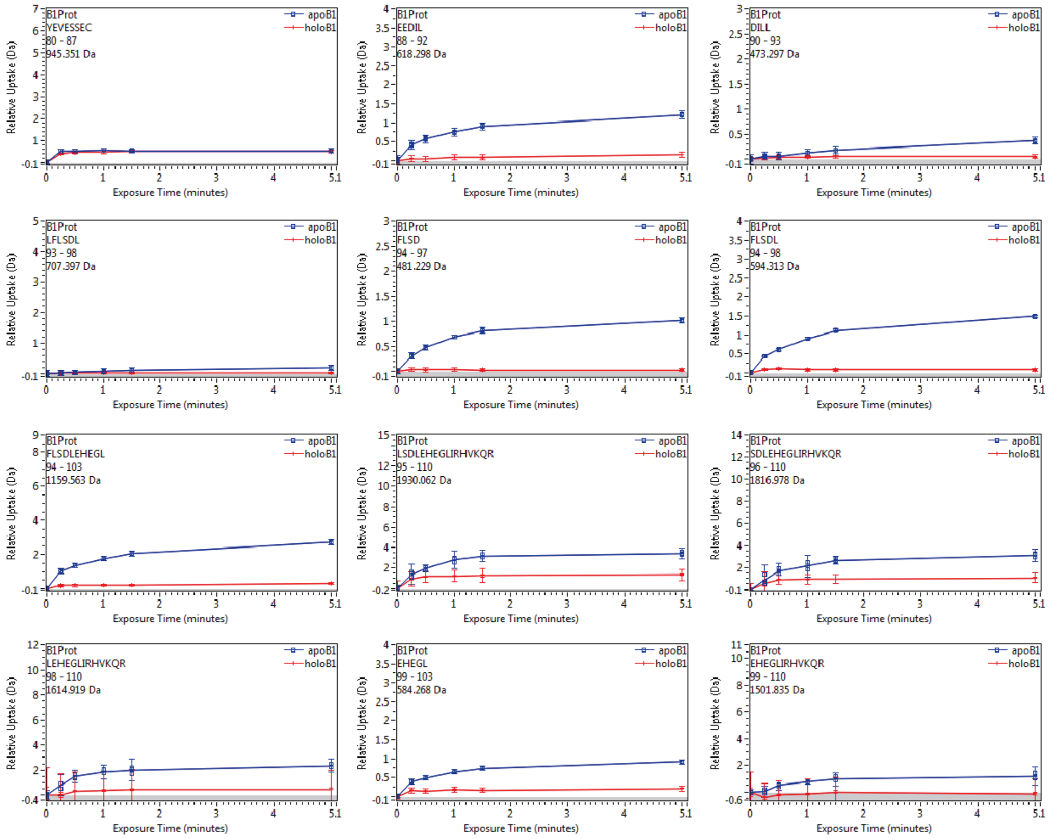

Supplementary Figure S6, cont. (a) Differential hydrogen-deuterium exchange, mapped onto peptic peptides of PadeB1. A color guide for relative fractional uptake is shown below. (b) Kinetics of deuterium uptake for PadeB1 regions showing significant differences in leader peptidebound-PadeB1 (red) *versus* free PadeB1 (blue). Error bars represent mean ± s.d. of triplicate measurements.

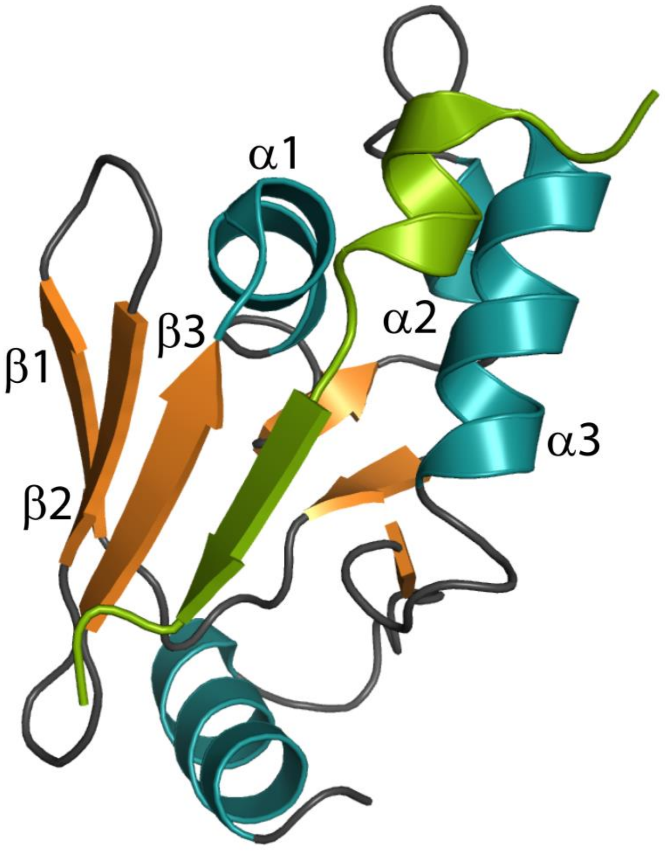

Supplemental Figure S7. Structure of a representative RRE with its leader peptide bound (PDB code 4V1T)[1]. The RRE belongs to LynD, a cyanobactin cyclodehydratase. Structural components that are not part of the RRE are hidden for clarity. Color code: leader peptide (green), β-strands of RRE (orange), α-helices of RRE (teal), loops of RRE (grey). The RRE of the lantibiotic dehydratase NisB (PDB code 4WD9) also binds its leader peptide in a similar manner[2].

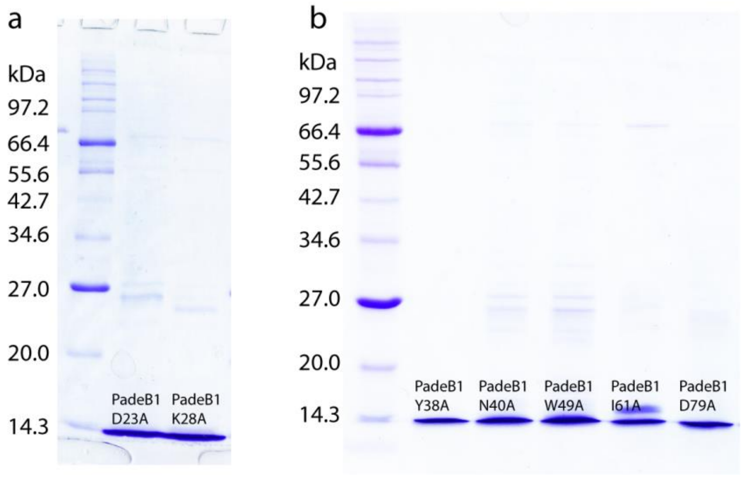

Supplementary Figure S8. (a-b) SDS-PAGE gels of purified PadeB1 variants. The proteins were purified by Ni-NTA and size-exclusion chromatography.

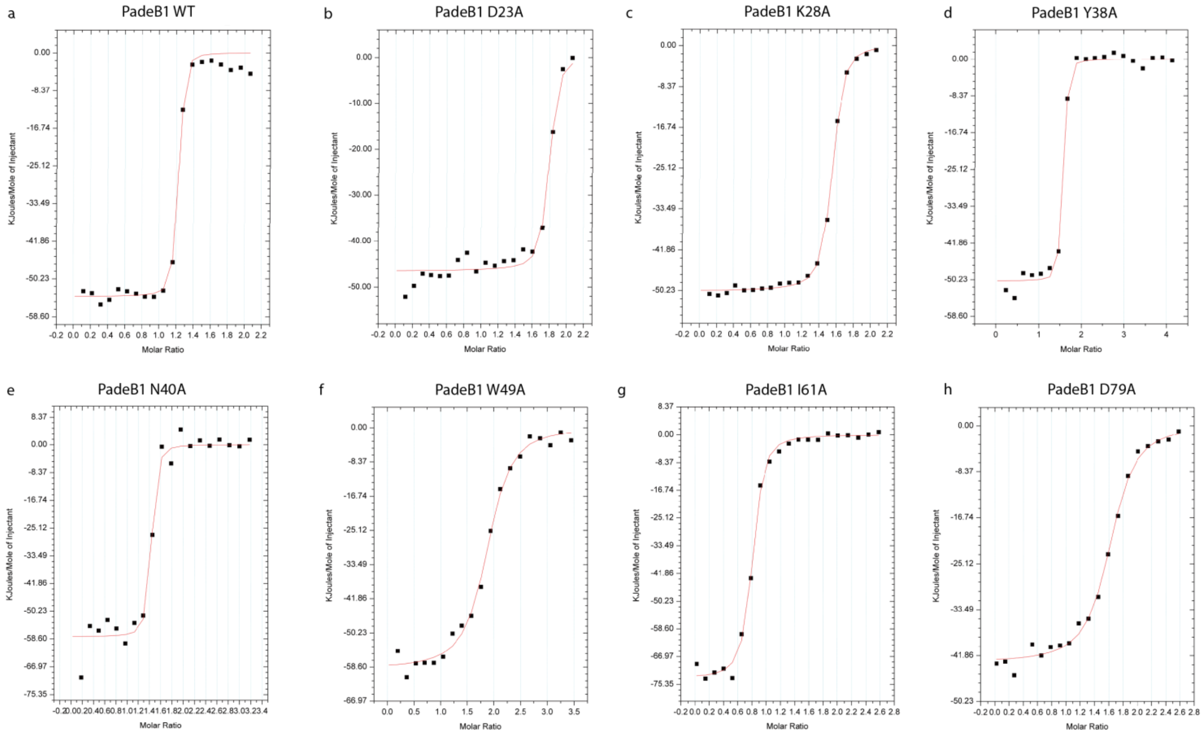

Supplementary Figure S9. (a-h) Representative binding curves for PadeB1 variants plus leader peptide. Data were fit to a "one set of sites" model (see Methods section). The first injection for each experiment was omitted from data analysis.

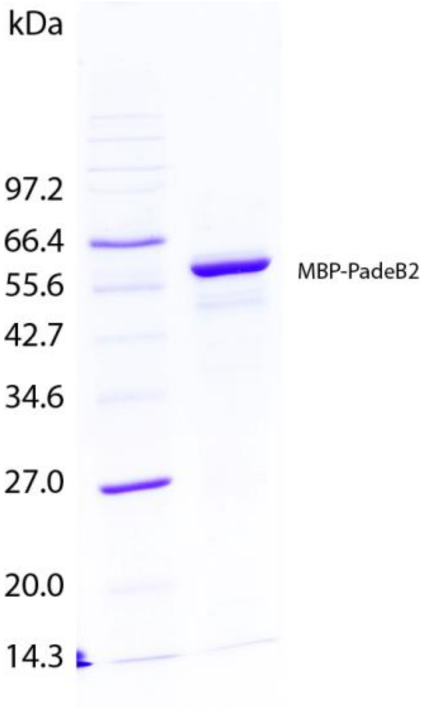

Supplementary Figure S10. SDS-PAGE gel of purified MBP-PadeB2. The protein was purified by Ni-NTA and size-exclusion chromatography.

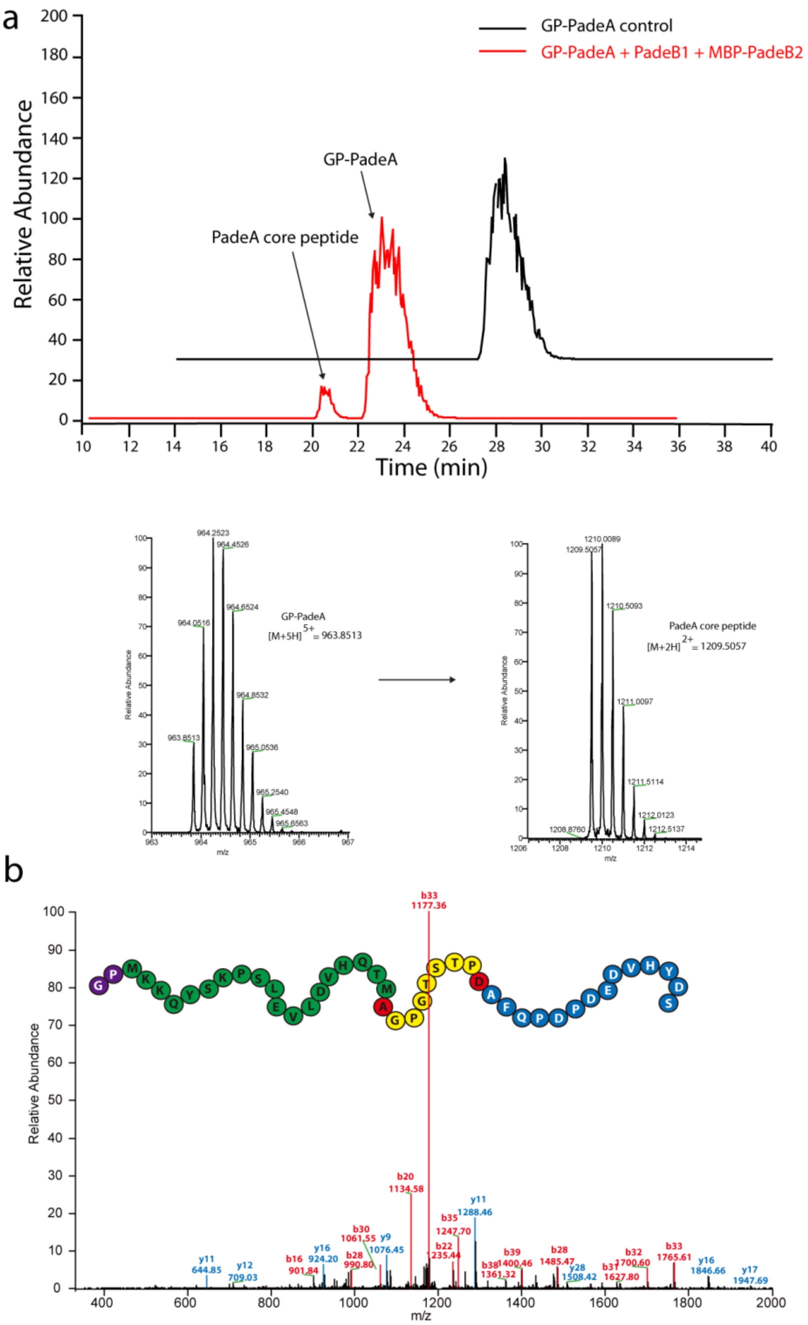

Supplementary Figure S11. (a) Extracted ion currents and mass spectra from the assay of PadeB1 and MBP-PadeB2 with GP-PadeA. The core peptide is only produced when both PadeB1 and MBP-PadeB2 are present. (b) MS^2 spectrum from the assay. The primary sequence of GP-PadeA is shown.

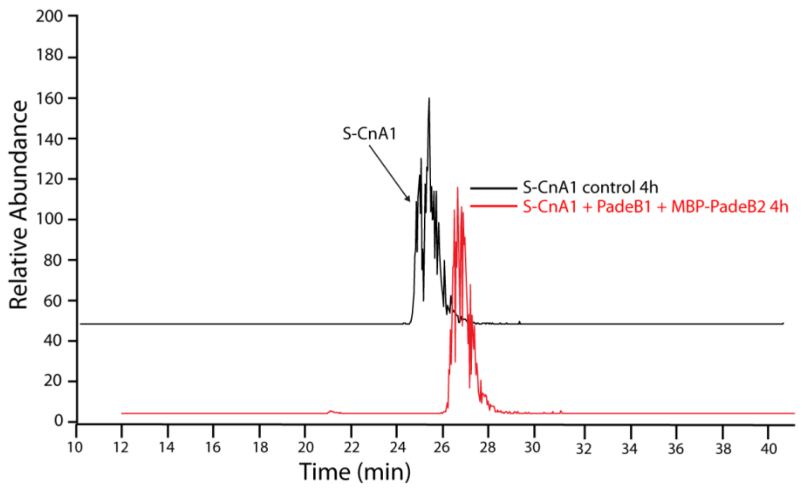

Supplementary Figure S12. Extracted ion currents from the assay of PadeB1 and MBP-PadeB2 with S-CnA1.

Supplementary References

1. Koehnke, J. *et al*. Structural analysis of leader peptide binding enables leader-free cyanobactin processing. *Nat. Chem. Biol.* **11,** 558-563 (2015).

2. Ortega, M. A. *et al*. Structure and tRNA specificity of MibB, a lantibiotic dehydratase from actinobacteria involved in NAI-107 biosynthesis. *Chem. Biol.* **23,** 370-380 (2016).
